# External validation of the CALLY index and development of a laboratory-based model for 28-day mortality in sepsis: a two-center study

**DOI:** 10.3389/fmed.2026.1875464

**Published:** 2026-09-10

**Authors:** Zhao-Yin Fu, Xing-Rong Yu, Yong-Ling Yang, Yu-Long Bai, Jia-Shan You, Ye-Zhao Li, Feng-Lan Qin, Zhi-Wei Huang

**Affiliations:** 1 Department of Critical Care Medicine, The First People’s Hospital of Qinzhou/Tenth Affiliated Hospital of Guangxi Medical University, Qinzhou, China; 2 Department of Emergency, The First Affiliated Hospital of Guangxi Medical University, Nanning, China

**Keywords:** CALLY index, machine learning, mortality, predictive model, sepsis

## Abstract

**Background:**

Early identification of high-risk sepsis patients remains challenging. The C-reactive protein-Albumin-Lymphocyte (CALLY) index, which integrates inflammation, nutritional status, and immune function, may hold prognostic value in this setting.

**Methods:**

We retrospectively analyzed sepsis patients from two Chinese hospitals (discovery cohort: *n* = 695; validation cohort: *n* = 515). Three machine learning algorithms were applied to identify key predictors. A logistic regression model was constructed, internally validated, and externally tested. Model performance was compared with APACHE II and SOFA scores using DeLong's test.

**Results:**

The non-survivors had markedly lower CALLY values (median 0.2 vs. 0.8, *P* < 0.001). CALLY, lactate dehydrogenase (LDH), total proteins (TP), and platelet count (PLT) were consistently selected as top predictors across all three machine learning methods. The final model demonstrated good discrimination in the development cohort (AUC = 0.877; 95% CI: 0.847–0.908), with a significantly higher AUC than APACHE II (*P* < 0.001) and SOFA (*P* = 0.001). Calibration was satisfactory (Hosmer–Lemeshow *P* = 0.638), and decision curve analysis indicated clinical net benefit. In external validation, the model retained predictive ability (AUC = 0.920), and CALLY remained significantly lower in non-survivors (*P* < 0.001).

**Conclusion:**

The CALLY index is an acceptable, readily available biomarker strongly associated with 28-day mortality in sepsis. A prediction model incorporating CALLY, LDH, TP, and PLT offers performance comparable or superior to conventional severity scores and may support early risk stratification in clinical practice, provided that calibration-in-the-large is updated when the model is applied to settings with substantially different baseline mortality.

## Introduction

Sepsis is defined as life-threatening organ dysfunction resulting from a dysregulated host response to infection (1). Despite progress in critical care, identifying patients at high risk of poor outcomes early in the clinical course continues to pose a challenge (2). Prognostic tools that rely on routinely available, inexpensive laboratory tests are particularly desirable, as they can facilitate timely intervention and more precise patient stratification (3).

Nutritional status and systemic inflammation are closely intertwined and both contribute substantially to the pathophysiology and prognosis of sepsis (4, 5). Serum albumin, a well-established marker of nutritional reserve, declines markedly during the acute-phase response, and hypoalbuminemia consistently predicts adverse outcomes (6). Lymphopenia, a hallmark of sepsis-induced immunosuppression, is likewise a strong predictor of mortality (7). C-reactive protein (CRP), a classic acute-phase reactant, provides a direct readout of systemic inflammatory intensity (8). Given the interplay among these three axes, nutrition (albumin), immune competence (lymphocyte count), and inflammation (CRP), a composite index that captures all three simultaneously may offer a more comprehensive prognostic assessment than any single parameter.

The C-reactive protein–Albumin–Lymphocyte (CALLY) index has recently been proposed as an integrative biomarker that mathematically combines CRP, albumin, and absolute lymphocyte count to provide a snapshot of physiological derangement in critical illness. Although the CALLY index has emerged as a promising prognostic marker in oncology and certain inflammatory conditions (9, 10), several important gaps remain. First, existing evidence is derived predominantly from single-center cohorts, and the generalizability of CALLY across independent centers with distinct case-mixes has not been externally validated. Second, no study has directly benchmarked CALLY against established severity scores. Third, CALLY has been evaluated almost exclusively as a standalone marker, whereas its incremental value when integrated with complementary biomarkers remains unexplored.

To address these gaps, the present study makes three contributions. First, we provide external validation of CALLY in an independent center with a distinct case-mix, assessing its transportability beyond the derivation setting. Second, we perform a head-to-head comparison of CALLY vs. SOFA and APACHE II. Third, we develop and validate a parsimonious machine-learning-derived model that combines CALLY with complementary biomarkers, selected via a three-algorithm consensus procedure, and demonstrate its added predictive value over conventional scores.

## Methods

### Study design and population

This retrospective cohort study was reported in accordance with the Transparent Reporting of a multivariable prediction model for Individual Prognosis Or Diagnosis (TRIPOD) checklist. The development cohort comprised adult patients (aged ≥18 years) diagnosed with sepsis upon admission to the First Affiliated Hospital of Guangxi Medical University between January 2020 and December 2023. An independent external validation cohort was assembled from sepsis patients admitted to the First People's Hospital of Qinzhou during the same period, using identical inclusion and exclusion criteria. Sepsis was defined according to the Sepsis-3.0 criteria (11), which requires a suspected or confirmed infection coupled with an acute increase of ≥2 points in the Sequential Organ Failure Assessment (SOFA) score.

### Inclusion and exclusion criteria

Inclusion criteria were: (1) patient's age ≥18 years; (2) established diagnosis of sepsis; and (3) availability of complete clinical data for baseline characterization within seven days of admission. Exclusion criteria included: (1) pregnancy or lactation; and (2) incomplete key data required for model construction. No patient was excluded on the basis of a hospital stay shorter than 48 h; in particular, patients who died within 48 h of admission were retained in the cohort to avoid survivorship bias.

### Data collection and definitions

Demographic data, clinical characteristics, comorbidities [including shock, heart failure, atrial fibrillation, and Acute Respiratory Distress Syndrome (ARDS)], and therapeutic interventions (such as corticosteroid and vasopressor use) were extracted from electronic medical records. The primary outcome was all-cause mortality within 28 days of sepsis diagnosis. The index date (time zero) was defined as the date on which the patient first met the Sepsis-3.0 criteria; follow-up began on the index date and ended at death or day 28, whichever occurred first. All candidate predictors were measured within the first 24 h after the index date. For patients diagnosed with sepsis at the time of hospital presentation, the index date coincided with the admission date; for patients in whom sepsis was recognized later (those transferred from other wards), the index date was the date of sepsis diagnosis. Disease severity was assessed using the Acute Physiology and Chronic Health Evaluation II (APACHE II) and SOFA scores, calculated using the worst values recorded within the first 24 h after the index date.

All laboratory parameters used as candidate predictors, including CRP, albumin, lymphocyte count, LDH, total protein, and platelet count, were obtained from blood samples drawn within the first 24 h after the index date (sepsis diagnosis). For patients already in the ICU at the time of sepsis diagnosis, samples were obtained within 24 h of diagnosis; for patients diagnosed on a general ward before ICU transfer, the first available blood sample after the sepsis diagnosis timestamp was used, regardless of subsequent ICU transfer. This rule anchors all predictor measurements to sepsis diagnosis rather than to ICU admission, minimizing heterogeneity in the physiological stage captured by the laboratory values. Of note, this rule reflects how the data were originally extracted from the electronic medical records—the first available blood sample drawn within 24 h of sepsis diagnosis was used for every patient—and therefore represents a clarification of that procedure rather than a re-extraction or re-analysis of the data. The C-reactive protein-Albumin-Lymphocyte index (CALLY index) was calculated for each patient using the following formula: CALLY = [Albumin [g/L] × Lymphocyte count [×10^9^/L]]/[CRP (mg/L)]. Of note, this formula does not apply the ×10^4^ scaling factor used in some published variants of the index; accordingly, absolute values and cut-offs reported here are not directly comparable to thresholds derived with scaled variants.

### Machine learning-based predictor selection

Three machine learning algorithms, including least absolute shrinkage and selection operator (LASSO) logistic regression, random forest (RF), and gradient boosting machine (GBM), were used exclusively as feature-selection tools to identify a parsimonious set of candidate predictors; the final prediction model was a conventional multivariable logistic regression fitted on the selected variables. The rationale for this two-stage approach is that machine learning algorithms can capture complex, potentially nonlinear relationships during the screening phase, whereas logistic regression offers interpretability and ease of clinical deployment. All preprocessing, feature selection, and hyperparameter tuning were performed exclusively within the training partition (70% of the development cohort) to avoid information leakage; the testing partition (30%) was not used at any stage of model construction. LASSO logistic regression was implemented via 10-fold cross-validation; the regularization parameter (*λ*) was selected as the value minimizing binomial deviance, and variables with non-zero coefficients were retained. The random forest classifier was trained with 500 trees, with predictor importance quantified by the mean decrease in Gini impurity; the top 10 variables were recorded. For RF, the number of trees was fixed at 500 and the number of variables randomly sampled at each split (mtry) was tuned over three candidate values (2, 21, and 41), with mtry = 2 selected by cross-validation. For GBM, hyperparameters were tuned over a grid of nine candidate combinations (number of trees: 50, 100, or 150; interaction depth: 1, 2, or 3; shrinkage: 0.1; minimum observations per terminal node: 10), from which cross-validation selected 100 trees, an interaction depth of 2, and a shrinkage of 0.1; variable importance was assessed by the relative influence score. In both algorithms, hyperparameter tuning was performed by five-fold cross-validation entirely within the 70% training partition, and discrimination was evaluated on the held-out 30% testing partition, which was not used at any stage of training or tuning. The development cohort retained all eligible patients (mortality 20.0%); class imbalance was not corrected by resampling, and discrimination was summarized by the AUC, which is insensitive to event prevalence, with thresholds selected by Youden's index. The top-10 threshold was pre-specified to balance comprehensiveness against over-selection given the candidate laboratory-variable pool and was fixed before the three-way intersection was examined. Only variables consistently ranked among the top predictors across all three models, appearing in the LASSO-selected set and within the top 10 of both RF and GBM, were included in the subsequent multivariable logistic regression. All machine learning procedures were implemented in R (version 4.3.1) using the “glmnet”, “randomForest”, and “gbm” packages.

### Model development and evaluation

A multivariate logistic regression model was constructed using the selected features to estimate the probability of 28-day all-cause mortality. The final model was visualized as a nomogram to facilitate clinical application. Model performance was evaluated by the Receiver Operating Characteristic (ROC) curve and the Area Under the Curve (AUC) with 95% confidence intervals (CI) estimated by DeLong's method in the training, internal testing, whole development, and external validation cohorts. Sensitivity, specificity, positive predictive value (PPV), and negative predictive value (NPV) were also used to assess the predictive value of the model. Internal validation of the final model was performed using bootstrap resampling (1,000 resamples) applied to the final four-variable logistic regression model after predictor selection, yielding an optimism-corrected C-index of 0.871; the machine-learning feature-selection procedure was performed once on the 70% training partition and was not repeated within each bootstrap resample. A C-index above 0.7 was considered to indicate good discrimination. Model robustness was evaluated using calibration curve with the Hosmer-Lemeshow goodness-of-fit test, a non-significant *P* value >0.05 indicates good calibration. Patients in the development cohort were stratified into high- and low-CALLY index groups based on the optimal cutoff value determined by Youden's index from receiver operating characteristic (ROC) curve analysis.

### Statistical analysis

Continuous variables were presented as median [interquartile range (IQR)] and compared using the Mann–Whitney *U* test, while categorical variables were expressed as numbers (percentages) and analyzed using the chi-square or Fisher's exact test, as appropriate. Multicollinearity among predictors was assessed using the variance inflation factor (VIF); no significant multicollinearity was observed (all VIF <5). A multivariable logistic regression model was developed using the training set (70% of the development cohort, selected via random sampling) and validated internally on the testing set (30%). The predictive performance of the final model was compared against the APACHE II and SOFA scores using DeLong's test for AUC comparison. The category-free (continuous) Net Reclassification Improvement (NRI) vs. SOFA was computed in the external validation cohort, defined as the sum of the proportion of events correctly reclassified upward minus the proportion reclassified downward, plus the proportion of non-events correctly reclassified downward minus the proportion reclassified upward. Predicted probabilities from the full model were compared with those from a logistic regression containing SOFA alone, and the 95% confidence interval was estimated from 2,000 bootstrap resamples; the event and non-event components are reported separately. To isolate the incremental contribution of the CALLY index itself, a nested category-free NRI was additionally computed by comparing the full four-variable model with a three-variable model excluding CALLY (LDH, TP, and PLT only), using the same continuous metric and bootstrap procedure. Calibration was summarized by the calibration intercept (calibration-in-the-large), the calibration slope, the observed-to-expected (O/E) ratio, and the Hosmer–Lemeshow test. Finally, the model's generalizability was assessed by applying it without coefficient re-estimation to the external validation cohort. All statistical analyses were performed using R software (version 4.3.1).

## Results

### Association between the CALLY index and mortality in sepsis patients

**Figure 1:**
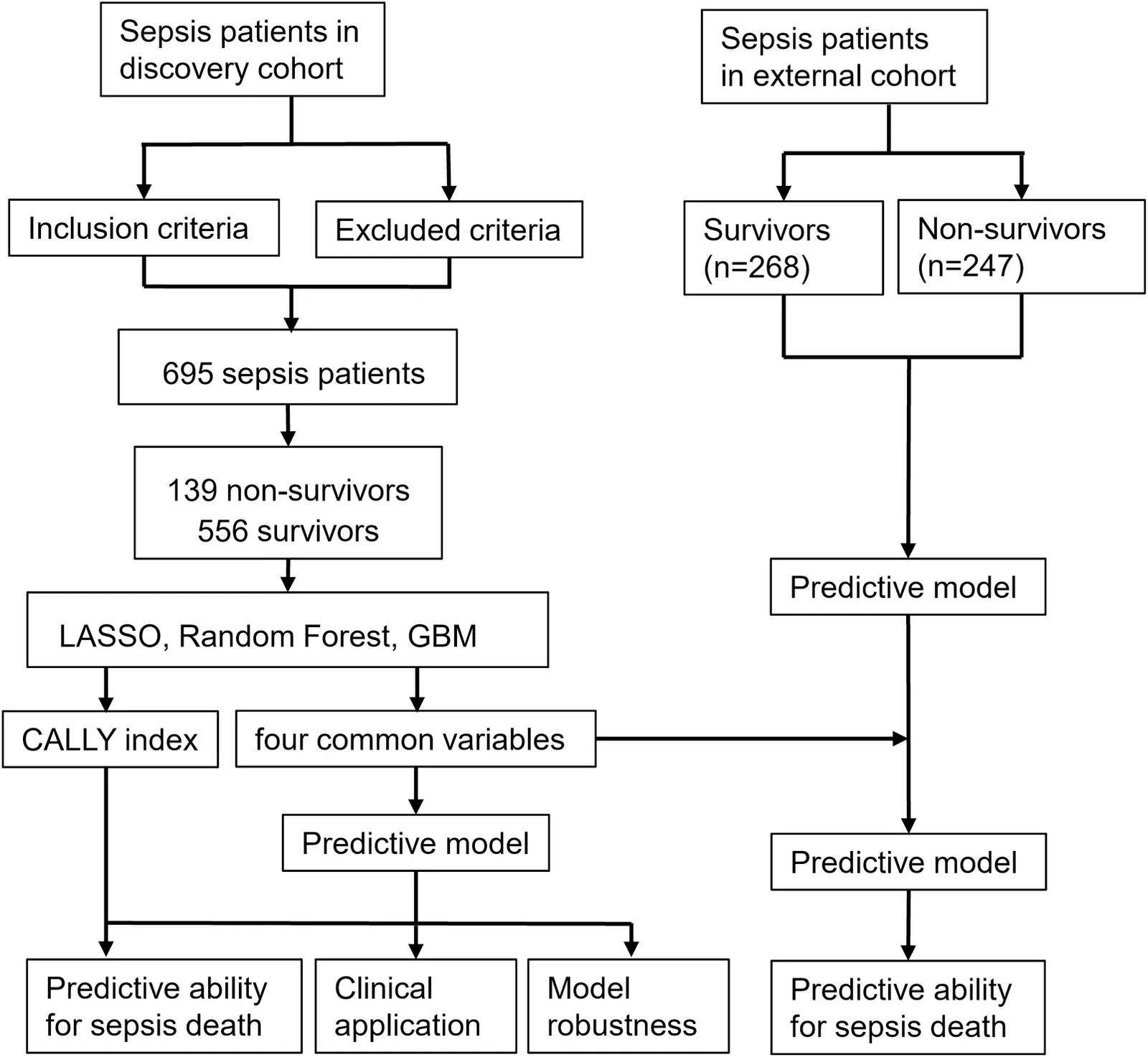
The study flowchart of present study.

The study flowchart is presented in Figure 1. The development cohort comprised 695 sepsis patients from the First Affiliated Hospital of Guangxi Medical University, including 556 survivors and 139 non-survivors within 28 days. Baseline comparisons revealed no significant differences between survivors and non-survivors regarding age, sex, BMI, smoking status, alcohol consumption, concomitant ARDS, corticosteroid use, or site of infection (all *P* > 0.05). In contrast, non-survivors exhibited significantly higher rates of concomitant shock, heart failure, atrial fibrillation, and vasopressor use (all *P* < 0.05). Furthermore, non-survivors had significantly higher APACHE II and SOFA scores (both *P* < 0.001).

Laboratory analyses demonstrated significant differences between the two groups across a broad spectrum of parameters, including complete blood counts, liver function tests, lipid profiles, coagulation function, and inflammatory markers (CRP, PLR, NLR) (all *P* < 0.05). Notably, the CALLY index was markedly lower in non-survivors [median 0.2 (IQR: 0.1–0.5)] compared to survivors [median 0.8 (IQR: 0.3–2.4)] (*P* < 0.001), as detailed in Table 1.

**Table 1:** Characteristics of included study.

Variables	Survivors (*N* = 556)	Non-survivors (*N* = 139)	*P* value
Gender			
Male	369 (66.4%)	105 (75.5%)	0.048
Female	187 (33.6%)	34 (24.5%)	
Age (years)	59.0 (49.0–69.0)	63.0 (45.0–74.0)	0.138
Smoking	222 (39.9%)	58 (41.7%)	0.772
BMI	23.0 (20.2–25.4)	23.1 (20.2–26.7)	0.175
Alcohol	211 (37.9%)	56 (40.3%)	0.682
Shock	262 (47.1%)	128 (92.1%)	<0.001
Heart failure	89 (16%)	39 (28.1%)	0.002
Atrial fibrillation	72 (12.9%)	32 (23%)	0.004
ARDS	65 (11.7%)	23 (16.5%)	0.162
Vasopressor use	328 (59%)	139 (100%)	<0.001
Corticosteroid use	342 (61.5%)	75 (54%)	0.126
TBIL	11.3 (7.1–24.0)	39.9 (18.1–128.8)	<0.001
APACHE II score	19.5 (17.5–21.5)	23.5 (21.0–24.5)	<0.001
SOFA score	8.0 (7.0–9.0)	15.0 (13.0–18.0)	<0.001
Infected sites			
blood	115 (20.7%)	22 (15.8%)	0.597
liver	105 (18.9%)	23 (16.5%)	
lung	112 (20.1%)	33 (23.7%)	
urinary	118 (21.2%)	31 (22.3%)	
others	106 (19.1%)	30 (21.6%)	
IBIL (μmoL/L)	6.0 (3.5–14.1)	26.5 (11.6–75.8)	<0.001
DBIL (μmoL/L)	5.0 (3.1–9.2)	9.6 (4.6–31.0)	<0.001
IBIL/DBIL ratio	0.6 (0.4–0.7)	0.7 (0.5–0.8)	<0.001
TP (g/L)	62.2 (55.5–68.5)	51.4 (44.1–57.6)	<0.001
ALB (g/L)	31.9 (28.4–35.2)	28.4 (24.7–32.2)	<0.001
GLB (g/L)	29.5 (24.8–34.9)	21.8 (17.5–27.9)	<0.001
A/G ratio	1.1 (0.9–1.3)	1.3 (1.0–1.7)	<0.001
rGT (μmol/L)	64.5 (38.0–127.0)	51.0 (31.5–117.5)	0.048
TBA (μmol/L)	5.3 (2.8–12.5)	16.5 (6.8–75.5)	<0.001
AST (U/L)	32.0 (21.0–52.5)	141.0 (41.0–530.5)	<0.001
ALT (U/L)	24.0 (12.0–48.0)	62.0 (18.0–278.5)	<0.001
AST/ALT ratio	1.4 (0.9–2.3)	2.8 (1.4–4.6)	<0.001
ALP (U/L)	109.0 (79.0–163.0)	128.0 (84.5–223.0)	0.006
PA (mg/L)	144.1 (88.8–204.0)	87.1 (51.1–120.9)	<0.001
ChE (U/L)	3,525.5 (2,521.0–5,117.0)	2,963.0 (2,071.0–4,416.5)	0.001
WBC ( × 10^9^/L)	8.8 (6.2–12.2)	13.1 (6.5–19.9)	<0.001
RBC ( × 1,0^12^/L)	3.0 (2.6–3.6)	2.4 (1.9–3.0)	<0.001
HB (g/L)	86.0 (71.4–99.0)	70.0 (57.1–89.5)	<0.001
PLT ( × 10^9^/L)	246.5 (124.8–361.1)	60.2 (30.2–117.0)	<0.001
NEU ( × 10^9^/L)	6.3 (4.1–9.9)	11.6 (5.8–18.2)	<0.001
LYM ( × 10^9^/L)	1.2 (0.8–1.8)	0.7 (0.4–1.2)	<0.001
MONO ( × 10^9^/L)	0.6 (0.4–0.9)	0.4 (0.2–1.1)	0.001
CRP (mg/L)	48.3 (15.5–111.5)	110.7 (62.8–173.6)	<0.001
CKMB (U/L)	11.5 (8.0–18.0)	24.0 (15.5–76.0)	<0.001
LDH (U/L)	259.0 (192.0–368.0)	591.0 (302.0–1,142.5)	<0.001
CKMB-CK (U/L)	0.2 (0.1–0.4)	0.1 (0.0–0.3)	<0.001
APTT (s)	32.8 (29.9–37.0)	43.6 (35.3–55.9)	<0.001
PT (s)	13.0 (11.9–14.9)	17.4 (14.1–28.2)	<0.001
FIB (g/L)	4.0 (3.0–4.9)	2.9 (1.8–4.5)	<0.001
TT (s)	12.3 (11.2–13.8)	14.0 (11.6–17.3)	<0.001
INR	1.1 (1.0–1.3)	1.5 (1.2–2.5)	<0.001
TCHO (mmol/L)	3.2 (2.3–4.2)	2.7 (1.8–3.7)	<0.001
TG (mmol/L)	1.6 (1.1–2.4)	1.8 (1.0–2.5)	0.949
HDLC (mmol/L)	0.7 (0.5–0.9)	0.6 (0.4–0.9)	0.001
LDLC (mmol/L)	1.7 (1.1–2.5)	1.4 (0.8–1.9)	<0.001
PLR	200.0 (122.3–309.7)	79.1 (29.1–196.6)	<0.001
NLR	5.2 (2.8–10.8)	15.9 (6.2–23.9)	<0.001
Cally index	0.8 (0.3–2.4)	0.2 (0.1–0.5)	<0.001

### Machine learning-based identification of key predictors

**Figure 2:**
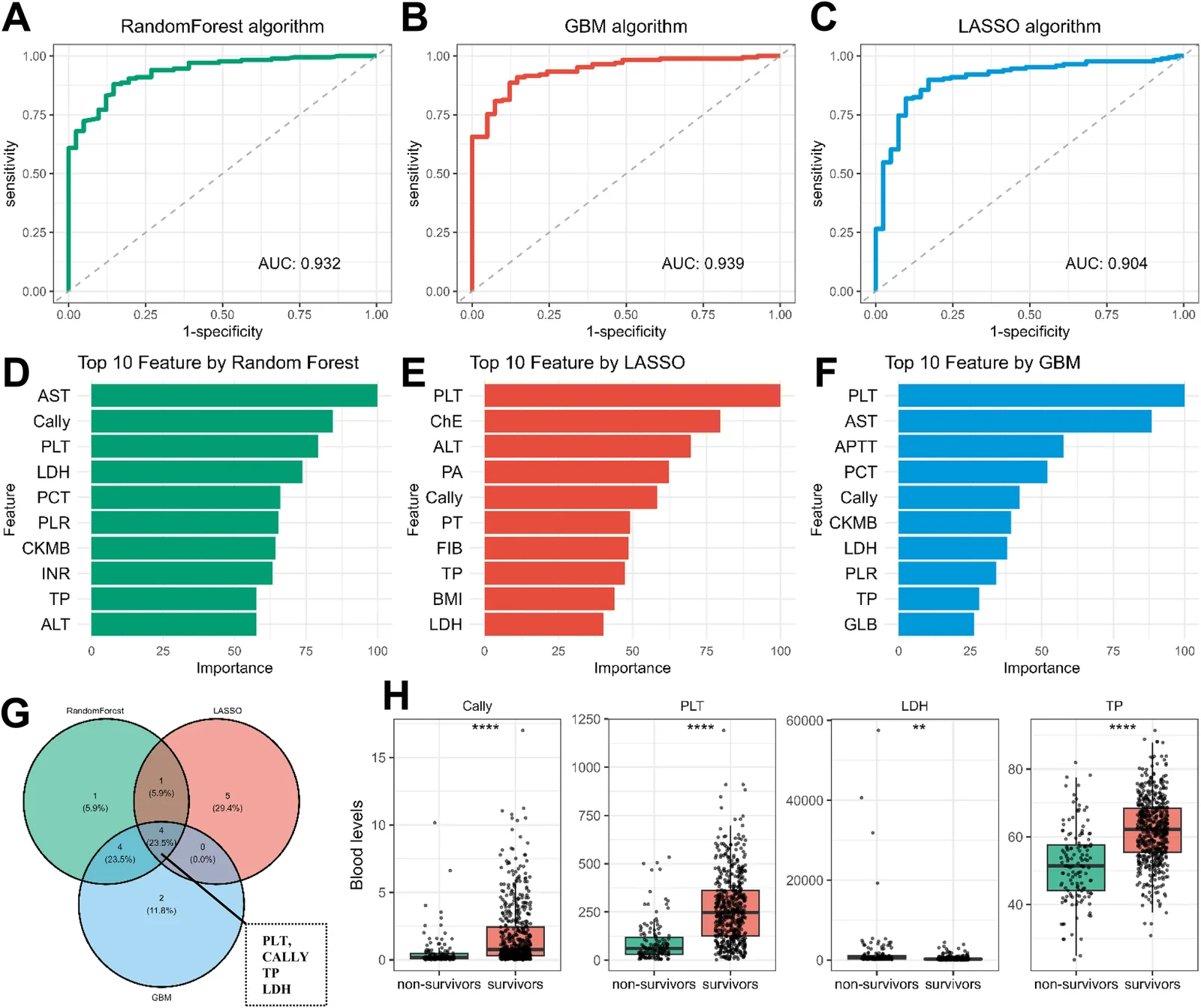
Identification of key predictors for mortality in sepsis patients. **(A–C)** Predictive value of non-survivors of sepsis by **(A)** Random Forest algorithm, **(B)** GBM algorithm and **(C)** LASSO algorithm; **(D–F)** top ten variables screened by **(D)** Random Forest algorithm, **(E)** LASSO algorithm and **(F)** GBM algorithm; **(G)** Venn plot for the common variables of Random Forest model, GBM algorithm and LASSO algorithm; The four variables shared by LASSO and GBM (PLT, CALLY, TP, LDH) were also selected by Random Forest and thus appear in the central three-way intersection (*n* = 4); consequently the LASSO∩GBM-only region equals 0. **(H)** Comparison of four variables levels between non-survivors and survival sepsis patients. *p < 0.05, **p < 0.01, ***p < 0.001, ****p < 0.0001.

To systematically identify laboratory predictors of 28-day mortality, we employed three machine learning algorithms: LASSO regression, Random Forest, and Gradient Boosting Machine (GBM). All models demonstrated good discriminatory ability on the held-out testing partition (30% of the development cohort; LASSO AUC = 0.904; Random Forest AUC = 0.932; GBM AUC = 0.939; Figures 2A–C). The intersection of the top 10 most important variables from each algorithm identified four common predictors: the CALLY index, lactate dehydrogenase (LDH), total proteins (TP), and platelet count (PLT) (Figures 2D–G). Consistent with univariate analysis, non-survivors exhibited significantly elevated levels of LDH, while their CALLY index, TP and PLT levels were significantly reduced (all *P* < 0.05; Figure 2H).

### Predictive performance and clinical correlates of the CALLY index

**Figure 3:**
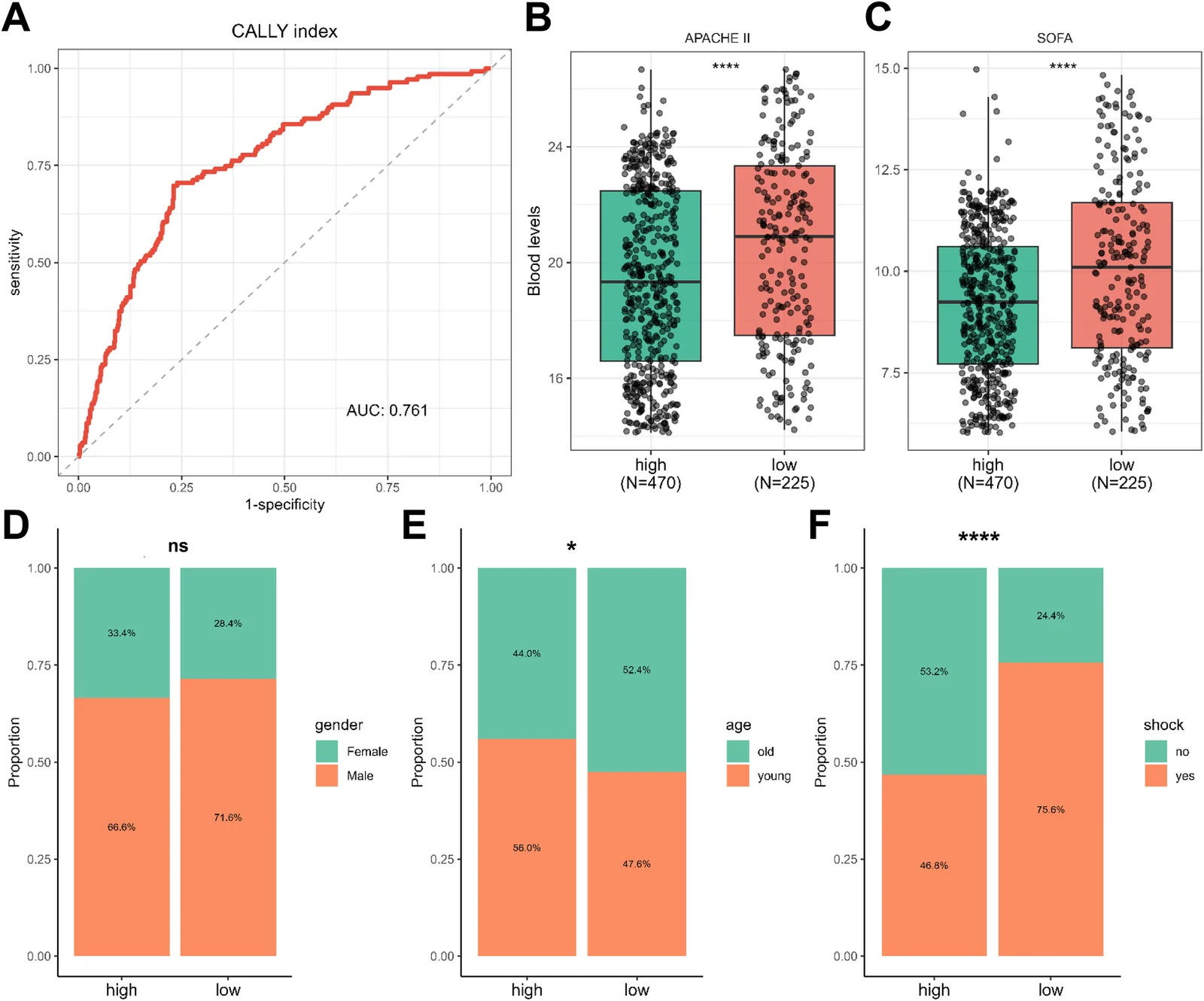
Predictive performance and clinical correlates of the CALLY index. **(A)** Predictive value of the CALLY index for 28-day mortality in sepsis patients; **(B,C)** Comparison of APACHE II score and SOFA score between high- and low- CALLY index group; **(D–F)** Comparison of patients’ gender, age and shock between high- and low- CALLY index group. *p < 0.05, **p < 0.01, ***p < 0.001, ****p < 0.0001.

ROC curve analysis indicated that the CALLY index alone has moderate predictive value for 28-day mortality, with an AUC of 0.761 (95% CI: 0.717–0.805; Figure 3A). The optimal cutoff value, determined by Youden's index, was 0.278, yielding a sensitivity of 69.8% (95% CI: 61.9%–77.0%) and a specificity of 77.0% (95% CI: 73.4%–80.4%). Using this threshold, all 695 patients were stratified into high- and low-CALLY index groups. This stratification revealed no significant difference in sex distribution (*P* > 0.05). However, patients in the low-CALLY index group were significantly older, had a higher prevalence of concomitant shock, and exhibited significantly higher APACHE II and SOFA scores (all *P* < 0.05; Figures 3B–F), indicating a strong association between a low CALLY index and 28-day mortality. Finally, after adjusting for key clinical confounders, including gender, age, heart failure, infected site, APACHE II score, and SOFA score, the CALLY index remained independently associated with 28-day mortality (Supplementary Table S1).

### Development and internal validation of a multivariable prediction model

**Figure 4:**
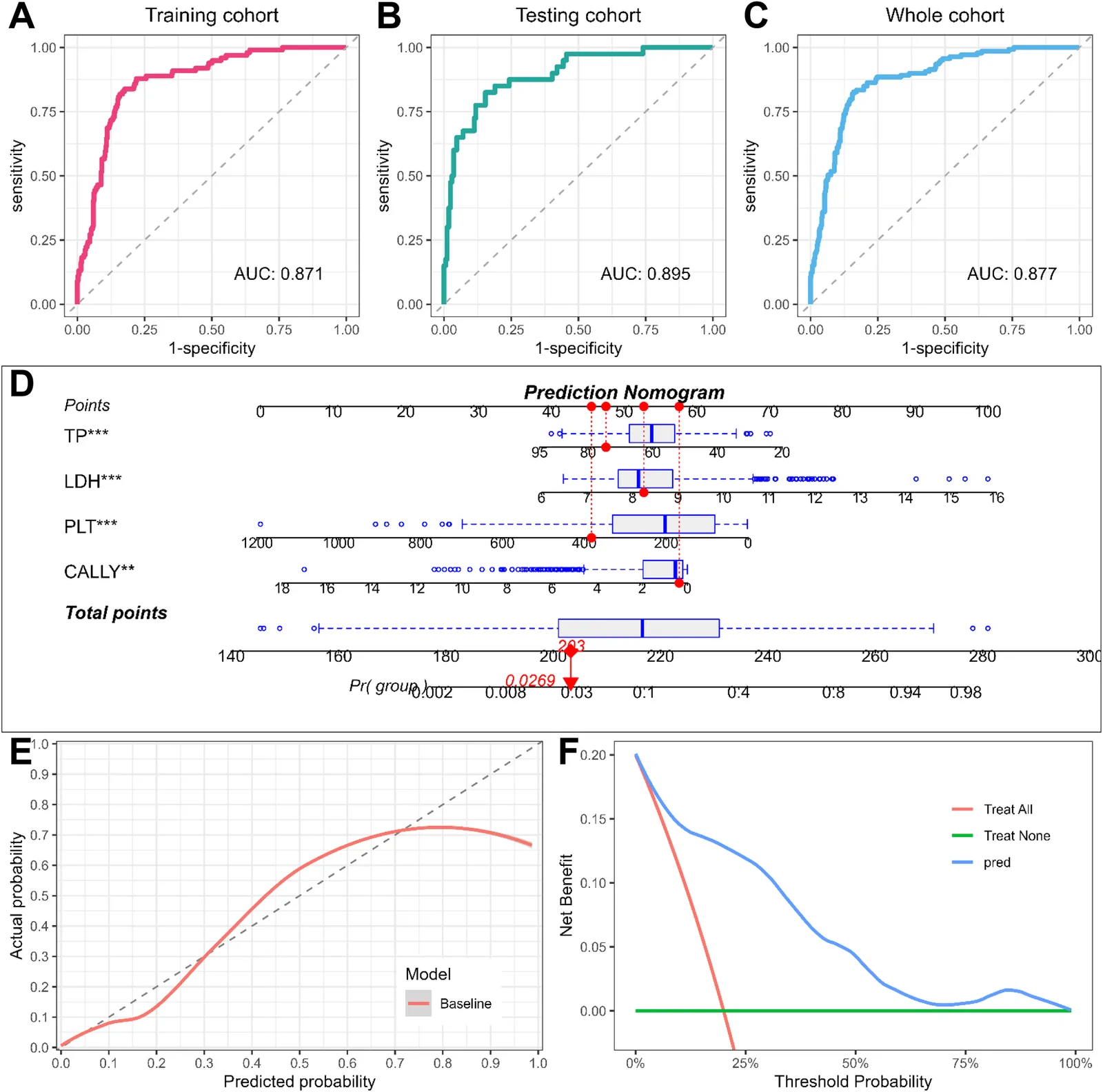
Development and internal validation of a multivariable prediction model. **(A–C)** Predictive value of the model for the non-survivors of sepsis in **(A)** Training cohort, **(B)** testing cohort, and the **(C)** whole cohort; **(D)** nomogram for the predictive model and clinical parameters. Each independent predictor of Nomogram was mapped to a “points” value at the top of the nomogram; **(E)** calibration curve for the nomogram was used to evaluate the consistency between model predicted probabilities and actual observed probabilities. **(F)** Decision curve Analysis (DCA) assess the net benefit of the nomogram across different high-risk thresholds.

We randomly split the development cohort into training (*n* = 486) and testing (*n* = 209) sets at a 7:3 ratio. A multivariable logistic regression model was constructed using the four key predictors identified by machine learning (CALLY index, LDH, TP, PLT). This model demonstrated high predictive accuracy across the training, internal testing, and whole development cohorts, with AUCs of 0.871 (95% CI: 0.834–0.908), 0.895 (95% CI: 0.839–0.951), and 0.877 (95% CI: 0.847–0.908), respectively (Figures 4A–C). Supplementary Table S2 reports the full multivariate regression results; all four predictors were independently associated with 28-day mortality, with CALLY (OR: 0.73, 95% CI: 0.59–0.90), PLT (OR: 0.99, 95% CI: 0.99–1.00), and TP (OR: 0.96, 95% CI: 0.93–0.98) inversely associated and LDH positively associated [OR: 1.89, 95% CI: 1.55–2.30, per one-unit increase in log2(LDH + 1)], consistent with the univariate analyses. The final prediction equation is logit(*P*) = −3.159 − 0.315 × CALLY + 0.636 × log2(LDH + 1) − 0.045 × TP − 0.006 × *P*LT, where *P* is the probability of 28-day mortality, CALLY is the unscaled CALLY index, LDH is serum lactate dehydrogenase in U/L entered after a log2(LDH + 1) transformation, TP is serum total protein in g/L, and PLT is the platelet count (×10^9^/L).

A corresponding nomogram was developed to facilitate clinical application (Figure 4D). The model showed strong overall performance, with an optimism-corrected C-index of 0.871, and a Brier score of 0.106. Calibration curves confirmed acceptable agreement between predicted and observed probabilities in the internal testing cohort (Hosmer–Lemeshow test *P* = 0.638; calibration intercept = −0.05; calibration slope = 1.09; observed-to-expected ratio = 0.94; Figure 4E). Decision curve analysis (DCA) further demonstrated that the model provides substantial net clinical benefit across a wide range of threshold probabilities (Figure 4F).

### Comparison of predictive performance with established scoring systems

**Figure 5:**
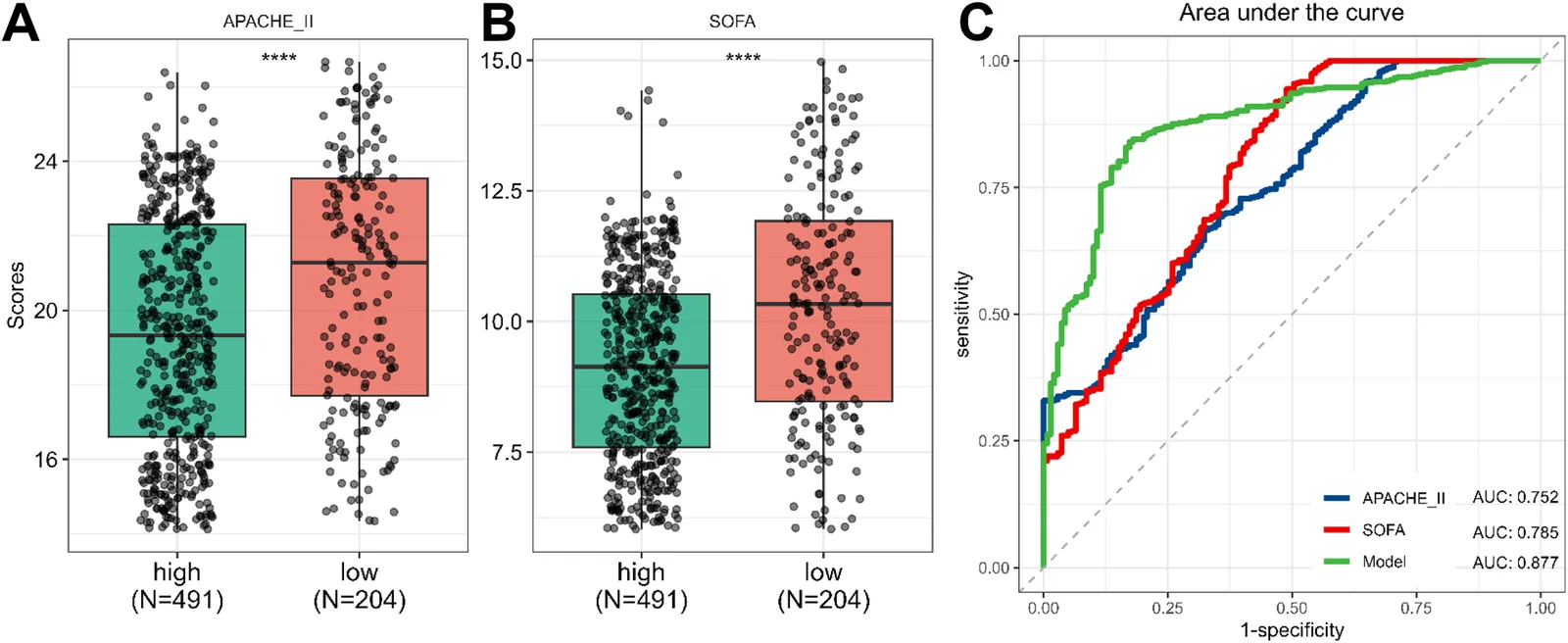
Comparison of predictive performance with established scoring systems. **(A,B)** Comparison of APACHE II score and SOFA score between high- and low- risk predictive group; **(C)** Comparison of the predictive value of the predictive model, APACHE II score and SOFA score for 28-day mortality in sepsis patients.

Using the model's optimal probability cutoff of 0.262, patients were classified into high- and low-risk groups. The high-risk group had significantly higher APACHE II and SOFA scores than the low-risk group (both *P* < 0.001; Figures 5A,B). DeLong's test revealed that our prediction model showed a higher AUC than both the APACHE II score (0.877 vs. 0.752, *P* < 0.001) and the SOFA score (0.877 vs. 0.785, *P* = 0.001), with statistically significant differences (Figure 5C).

### External validation of the prediction model

**Figure 6:**
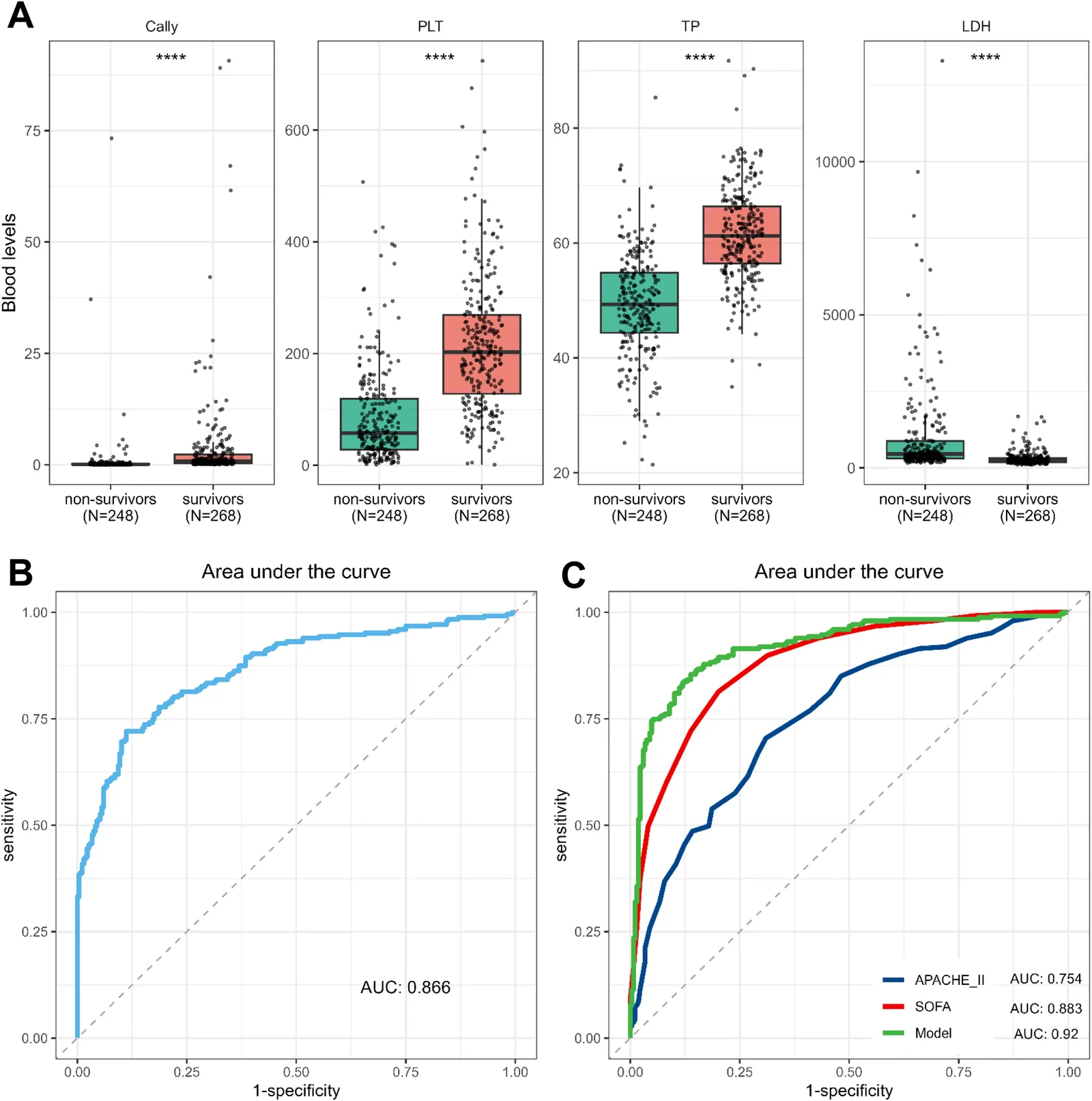
External validation of the prediction model. **(A)** Comparison of CALLY index, TP, LDH, PLT in survivors and non-survivors; **(B)** Predictive value of CALLY index on the 28-day mortality in sepsis patients; **(C)** Comparison of the predictive value of the predictive model, APACHE II score and SOFA score for 28-day mortality in sepsis patients.

To evaluate generalizability, we used an independent external validation cohort of 515 sepsis patients (268 survivors, 247 non-survivors) from another center. Baseline characteristics of the two cohorts are compared in Supplementary Table S3. In the external cohort, the CALLY index, PLT and TP was significantly lower in non-survivors, while LDH was higher in non-survivors (*P* < 0.001; Figure 6A). The CALLY index maintained acceptable discrimination, with an AUC of 0.866 (95% CI: 0.835–0.898; Figure 6B). The multivariate logistic regression analysis revealed that, after adjusting for key clinical confounders, including gender, age, vasopressor use, mechanical ventilation, APACHE II score, and SOFA score, the CALLY index remained independently associated with 28-day mortality (Supplementary Table S4).

We then applied the prediction model, without coefficient re-estimation, to the external validation cohort. The model showed good predictive performance, with an AUC of 0.920 (95% CI: 0.895–0.945), while the predictive value of the APACHE II and SOFA scores was 0.754 (95% CI: 0.713–0.796) and 0.883 (95% CI: 0.854–0.911), respectively, in the external cohort. DeLong's test indicated that the AUC of the model was significantly higher than that of APACHE II (*P* < 0.001) and higher than that of SOFA (*P* = 0.020) (Figure 6C). In the external cohort, the calibration intercept was 1.55 and the calibration slope was 1.18, with an observed-to-expected ratio of 1.71 (Supplementary Figure S1A). This calibration-in-the-large offset indicates that the model underestimated the absolute mortality risk in the external cohort, which is expected given its substantially higher baseline severity (28-day mortality 48% vs. 20% in the development cohort); discrimination remained high (AUC = 0.920), and the calibration slope close to unity indicates that the relative ranking of predicted risks was preserved. External DCA showed net benefit across clinically relevant thresholds (Supplementary Figure S1B), and the category-free NRI of the combined four-variable model vs. SOFA was 0.303 (95% CI: 0.138–0.463; *P* < 0.05), with event and non-event components of 0.206 and 0.097, respectively. In a nested comparison, adding the CALLY index to the model containing LDH, TP, and PLT yielded a category-free NRI of 0.492 (95% CI: 0.363–0.608), with event and non-event components of 0.895 and −0.403, respectively, indicating that the CALLY index itself contributed incremental reclassification beyond the other three laboratory markers.

## Discussion

Our study demonstrates that the CALLY index, a novel composite biomarker integrating key elements of nutritional status, systemic inflammation, and immune competence, is independently associated with 28-day mortality in patients with sepsis. Leveraging a robust methodology that combined conventional statistics with machine learning, we not only validated the prognostic value of the CALLY index but also developed and internally/externally validated a practical prediction model whose predictive performance exceeded that of established severity scores such as APACHE II and SOFA in the development setting.

It is important to note that hypoalbuminemia in sepsis is predominantly driven by IL-6/TNF-α-mediated suppression of hepatic synthesis rather than malnutrition *per se* (12, 13). Thus, CALLY index is presented as integrating inflammatory and immune information, with the nutritional component explicitly flagged as a secondary interpretation requiring caution. A low CALLY index reflects a triad of poor prognosis. This finding aligns with the growing body of evidence supporting the concept of “immunometabolism,” where metabolic derangements directly influence immune cell function and vice versa (14, 15). Our results suggest that the CALLY index serves as a simple, single-value surrogate for this complex biological crosstalk, offering a more holistic view of patient vulnerability than its individual components.

The prognostic value of the CALLY index in sepsis has been corroborated by several recent investigations. Yilmaz et al. (16) reported an AUC of 0.906 for 30-day mortality in 669 emergency department sepsis patients. Saridaş et al. (17) reported in a cohort of 1,644 patients, found that the CALLY index ranked as the top predictor by gain score and SHAP analysis across five machine learning models. Zhang et al. (18) further demonstrated independent associations between the CALLY index and 30-day mortality, 60-day mortality in 1,123 critically ill sepsis patients. Our findings, drawn from two Chinese centers, extend this evidence base by providing external validation and direct head-to-head comparison with established severity scores. A direct comparison reveals that the AUC of the CALLY index alone in our development cohort (0.761) is lower than the 0.906 reported by Yilmaz et al. (16) This discrepancy may stem from differences in study endpoints (28-day vs. 30-day mortality), patient demographics, or sepsis severity. Nevertheless, our study uniquely demonstrates that by integrating the CALLY index with other routine markers (LDH, TP, PLT), the predictive performance can be substantially enhanced.

The incremental value of adding the CALLY index to clinical models has been demonstrated across diverse contexts. Lin et al. (19) developed the “SOFAplusCALLY” model in septic shock patients, achieving an AUC of 0.90. Zhou et al. (20) showed that incorporating the CALLY index into a base clinical model improved the AUC for predicting post-ureteroscopic lithotripsy sepsis, with net reclassification improvement of 0.404. Fan et al. (21) reported that the CALLY index achieved superior prediction of multiple organ failure syndrome compared with APACHE II and SOFA in elderly sepsis patients. These convergent findings support the integration of nutritional–inflammatory indices into existing risk-stratification frameworks.

The application of multiple machine learning algorithms was a key strength of our study. The consistent identification of the CALLY index, alongside LDH, TP, and PLT, as top predictors by LASSO, Random Forest, and GBM models reinforces their biological and clinical relevance. LDH is a marker of cellular damage and turnover, TP reflects nutrition status of sepsis patients, and PLT count is linked to both inflammation and microthrombosis. The fact that our final logistic regression model, built on these four readily available laboratory parameters, achieved high discriminative accuracy (AUC > 0.85) in the development cohort and demonstrated good calibration and net clinical benefit highlights its potential utility in real-world clinical settings for early risk stratification.

Notably, our model demonstrated significantly better performance than the widely used APACHE II and SOFA scores in the development cohort. While these traditional scores are comprehensive, they require numerous physiological and laboratory variables that can be time-consuming to collect and calculate (22, 23). In contrast, our model relies solely on routine, inexpensive, and rapidly available blood tests, making it a more accessible tool, especially in resource-limited environments. In the external validation, the model retained robust discrimination (AUC: 0.920), numerically superior to the SOFA score (AUC: 0.883), further confirming its generalizability across different patient populations; however, given the calibration-in-the-large offset in the external cohort (observed-to-expected ratio: 1.71), calibration should be updated before the model is used clinically in settings with substantially different baseline mortality.

In this study, we confirmed the prognostic value of the CALLY index for 28-day all-cause mortality in critically ill patients and, importantly, extended the existing literature in three respects. First, whereas previous reports of CALLY were confined to single-center populations, we externally validated the index in an independent center with a distinct case-mix, supporting its transportability across settings. Second, unlike prior studies that assessed CALLY only by discriminative accuracy, we directly compared it with SOFA and APACHE II using NRI and DCA, demonstrating that the combined four-variable model provided incremental reclassification and net clinical benefit beyond conventional severity scores; a nested analysis further showed that the CALLY index itself contributed significant incremental reclassification when added to the other three markers (category-free NRI 0.492). Third, we moved beyond evaluating CALLY in isolation by constructing a parsimonious, machine-learning-derived model integrating CALLY with LDH, total protein, and platelet count and showed that this combination outperformed both CALLY alone and established scores. Together, these findings position CALLY not merely as another prognostic marker, but as a transportable, benchmarked, and integrable component of risk stratification in critical care.

This study has several limitations. Its retrospective design, despite its large sample size, introduces the potential for selection bias. Second, data on detailed nutritional support protocols and longitudinal changes in the CALLY index during hospitalization were not available, which limits our ability to explore its role in monitoring treatment response. Third, the model was constructed using only baseline laboratory values obtained within the first 24 h of admission. We did not evaluate the dynamic changes in these biomarkers, which could provide additional prognostic information. Fourth, despite successful external validation, notable differences existed between the two cohorts. For instance, patients in the external validation cohort had markedly higher rates of mechanical ventilation and hemodialysis, suggesting a higher baseline severity. This is reflected in their higher APACHE II and SOFA scores. While our model still performed well, these discrepancies highlight the need for further validation in more diverse, and ideally prospective, multicenter settings. In addition, the model underestimated the absolute mortality risk in the external cohort (observed-to-expected ratio: 1.71), reflecting the higher baseline severity of that population; although discrimination and relative risk ordering were preserved, calibration-in-the-large should be updated before the model is applied to settings with substantially different baseline mortality. In addition, the optimism-corrected C-index was estimated for the final four-variable model after predictor selection and therefore does not account for the optimism and uncertainty associated with the feature-selection procedure itself. Finally, serum lactate, although central to the Sepsis-3 framework, was not included among candidate predictors because it was not routinely measured within the first 24 h in >50% of patients during the study period, reflecting non-uniform testing protocols across centers. Future prospective, multicenter studies are warranted to confirm our findings and to investigate whether CALLY-guided therapeutic strategies can improve survival in sepsis patients.

## Conclusions

This study demonstrated that the CALLY index is an acceptable, integrative prognostic biomarker for mortality in sepsis patients. Our machine learning analyses further confirmed the incremental predictive value of the combined model, showing that models incorporating CALLY, LDH, TP, and PLT offer good discriminative value beyond the established severity scores, and may support early risk stratification in clinical practice; however, given the calibration-in-the-large offset observed in the external cohort, calibration should be updated before the model is used in settings with substantially different baseline mortality.

## Data Availability

The original contributions presented in the study are included in the article/Supplementary Material, further inquiries can be directed to the corresponding author.
