# Supplementary material for: External validation of the CALLY index and development of a laboratory-based model for 28-day mortality in sepsis: a two-center study
Source: Front Med (Lausanne). 2026 Sep 10;13:1875464. doi: 10.3389/fmed.2026.1875464 (PMC13601224; doi:10.3389/fmed.2026.1875464)
Supplement: Supplementary file 1 [file Datasheet1.docx]

**Supplement file**

Table S1 Univariate and Multivariate logistic regression analysis in the training cohort

| Terms | OR (Univariate) | OR (Multivariate) |
| --- | --- | --- |
| Gender | 0.64 (0.42-0.98, p = 0.039) | 0.90 (0.48-1.69, p =0.743) |
| Age | 1.01 (1.00-1.02, p = 0.134) | 0.99 (0.98-1.01, p =0.550) |
| Heart failure | 2.05 (1.33-3.16, p = 0.001) | 1.19 (0.62-2.29, p =0.609) |
| APACHE II score | 1.38 (1.29-1.49, p <0.001) | 1.40 (1.26-1.54, p <0.001) |
| SOFA score | 1.95 (1.71-2.21, p <0.001) | 1.96 (1.64-2.33, p <0.001) |
| Infected site | 0.96 (0.50-1.84, p = 0.911) |  |
|  | 1.12 (0.60-2.09, p = 0.726) |  |
|  | 1.48 (0.81-2.69, p = 0.203) |  |
|  | 1.44 (0.80-2.61, p = 0.228) |  |
| Cally | 0.51 (0.40-0.65, p <0.001) | 0.68 (0.50-0.91, p = 0.010) |
| PLT | 0.99 (0.99-0.99, p <0.001) | 0.99 (0.99-1.00, p <0.001) |
| LDH | 1.00 (1.00-1.00, p <0.001) | 1.00 (1.00-1.00, p <0.001) |
| TP | 0.90 (0.88-0.92, p <0.001) | 0.95 (0.92-0.98, p =0.004) |

Table S2 Multivariate logistic regression of the model

| Term | Coefficients | Std. Error | OR (95%CI) | P value |
| --- | --- | --- | --- | --- |
| Intercept | -3.159 | 1.189 | － | 0.007 |
| Cally | -0.314 | 0.109 | 0.73 (0.59-0.90) | 0.004 |
| PLT | -0.005 | 0.001 | 0.98 (0.97-1.00) | <0.001 |
| LDH | 0.635 | 0.100 | 1.89 (1.55-2.30) | <0.001 |
| TP | -0.045 | 0.013 | 0.96 (0.93-0.98) | <0.001 |

Table S3 Characteristics of external cohorts of sepsis patients

| Variables | Survivors (N=268) | Non-survivors (N=247) | P value |
| --- | --- | --- | --- |
| Age | 66.0 (55.5 to 76.0) | 69.0 (58.0 to 78.0) | 0.074 |
| Gender |  |  |  |
| Male | 157 (58.6%) | 151 (61.3%) | 0.591 |
| Female | 111 (41.4%) | 96 (38.7%) |  |
| Smoking | 74 (27.6%) | 70 (28.2%) | 0.954 |
| Hypertension | 134 (50%) | 119 (48%) | 0.712 |
| Diabetes | 85 (31.7%) | 77 (31%) | 0.945 |
| CHD | 53 (19.8%) | 67 (27%) | 0.066 |
| Trauma | 4 (1.5%) | 11 (4.4%) | 0.084 |
| Complications |  |  |  |
| COPD | 14 (5.2%) | 12 (4.8%) | 0.151 |
| Liver Failure | 2 (0.7%) | 5 (2%) |  |
| Surgery | 48 (17.9%) | 29 (11.7%) | 0.063 |
| Infected sites |  |  |  |
| ‌ abdomen‌ | 39 (14.6%) | 19 (7.7%) | <0.001 |
| Respiratory tract‌ | 163 (60.8%) | 181 (73%) |  |
| Urethra | 21 (7.8%) | 2 (0.8%) |  |
| Blood | 30 (11.2%) | 25 (10.1%) |  |
| Others | 15 (5.6%) | 21 (8.5%) |  |
| APACHE score | 24.0 (20.0 to 31.0) | 33.5 (27.0 to 41.0) | <0.001 |
| SOFA score | 10.0 (8.0 to 12.0) | 15.5 (13.0 to 18.0) | <0.001 |
| Mechanical ventilation | 212 (79.1%) | 247 (99.6%) | <0.001 |
| Hemodialysis | 30 (11.2%) | 89 (35.9%) | <0.001 |
| CRP (mg/L) | 53.8 (20.2 to 101.1) | 103.2 (59.4 to 165.5) | <0.001 |
| PCT (ng/ml) | 3.4 (0.4 to 16.0) | 10.0 (3.2 to 37.9) | <0.001 |
| WBC | 11.8 (9.2 to 15.4) | 18.7 (12.9 to 25.4) | <0.001 |
| NEU | 10.0 (7.4 to 13.1) | 16.7 (11.4 to 22.9) | <0.001 |
| LYM | 1.1 (0.8 to 1.7) | 0.4 (0.3 to 0.7) | <0.001 |
| PLT | 202.5 (127.0 to 269.5) | 57.5 (28.0 to 119.5) | <0.001 |
| RBC | 3.7 (3.1 to 4.4) | 2.2 (1.9 to 2.9) | <0.001 |
| TP | 61.2 (56.4 to 66.4) | 49.3 (44.3 to 54.8) | <0.001 |
| ALB | 33.3 (30.0 to 35.8) | 23.9 (20.8 to 26.9) | <0.001 |
| GLB | 29.7 (25.8 to 34.2) | 21.8 (17.9 to 27.7) | <0.001 |
| TBIL | 10.9 (7.4 to 18.6) | 30.4 (14.2 to 79.0) | <0.001 |
| IBIL | 4.9 (2.7 to 9.3) | 16.6 (6.5 to 47.5) | <0.001 |
| DBIL | 6.2 (3.9 to 9.9) | 13.3 (6.7 to 30.1) | <0.001 |
| ALT | 21.0 (14.0 to 45.5) | 54.0 (21.0 to 171.0) | <0.001 |
| AST | 30.0 (19.0 to 54.0) | 140.5 (51.0 to 417.5) | <0.001 |
| LDH | 242.5 (183.0 to 321.0) | 451.0 (303.5 to 878.0) | <0.001 |
| CCr | 96.4 (66.5 to 167.5) | 204.5 (119.2 to 375.9) | <0.001 |
| BUN | 9.0 (6.2 to 13.9) | 19.0 (11.3 to 29.4) | <0.001 |
| PT | 13.0 (11.9 to 14.9) | 19.0 (15.8 to 25.5) | <0.001 |
| APTT | 32.9 (28.8 to 38.8) | 50.7 (39.1 to 65.8) | <0.001 |
| INR | 1.1 (1.0 to 1.3) | 1.6 (1.3 to 2.3) | <0.001 |

Table S4 Univariable and multivariable logistic regression analysis in the external cohort

| Terms | OR (Univariate) | OR (Multivariate) |
| --- | --- | --- |
| Age | 1.01 (1.00-1.02, p = 0.095) | 1.05 (1.02-1.08, p = 0.004) |
| Gender | 0.89 (0.63-1.27, p = 0.531) |  |
| APACHE II score | 1.12 (1.09-1.14, p <0.001) | 0.98 (0.93-1.04, p = 0.504) |
| SOFA score | 1.69 (1.55-1.84, p <0.001) | 1.57 (1.29-1.91, p <0.001) |
| CRP | 1.01 (1.01-1.01, p <0.001) | 1.01 (1.00-1.02, p = 0.014) |
| LYM | 0.07 (0.04-0.11, p <0.001) | 0.12 (0.05-0.31, p <0.001) |
| PLT | 0.99 (0.98-0.99, p <0.001) | 1.00 (1.00-1.00, p = 0.773) |
| TP | 0.83 (0.81-0.86, p <0.001) | 0.98 (0.93-1.04, p = 0.597) |
| ALB | 0.66 (0.61-0.70, p <0.001) | 0.66 (0.57-0.76, p <0.001) |
| LDH | 1.00 (1.00-1.00, p <0.001) | 1.00 (1.00-1.00, p = 0.004) |
| Cally | 0.86 (0.79-0.94, p <0.001) | 1.06 (0.99-1.13, p = 0.097) |
|  |  |  |
| Mechanical ventilation | 65.25 (8.96-475.20, p <0.001) | 75.61 (3.96-1443.45, p = 0.004) |
| Vasopressor use | 24.59 (8.84-68.38, p <0.001) | 5.60 (0.94-33.34, p = 0.058) |

Figure S1 External cohort validation of a multivariable prediction model (A) Calibration curve for the nomogram was used to evaluate the consistency between model predicted probabilities and actual observed probabilities. (A) Decision curve Analysis (DCA) assess the net benefit of the nomogram across different high-risk thresholds.
